# Cytokine profiling of extracellular vesicles isolated from plasma in myalgic encephalomyelitis/chronic fatigue syndrome: a pilot study

**DOI:** 10.1186/s12967-020-02560-0

**Published:** 2020-10-12

**Authors:** Ludovic Giloteaux, Adam O’Neal, Jesús Castro-Marrero, Susan M. Levine, Maureen R. Hanson

**Affiliations:** 1 Department of Molecular Biology and Genetics, Cornell University, 323 Biotechnology Building, 526 Campus Road, Ithaca, NY 14853 USA; 2 CFS/ME Unit, Division of Rheumatology, Vall d’Hebron University Hospital Research Institute, Universitat Autònoma de Barcelona, Barcelona, 08035 Spain; 3 Private Practice, New York, NY USA

**Keywords:** Myalgic Encephalomyelitis/Chronic Fatigue Syndrome, Extracellular vesicles, Plasma, Cytokines

## Abstract

**Background:**

Myalgic Encephalomyelitis/Chronic Fatigue Syndrome (ME/CFS) is a debilitating disease of unknown etiology lasting for a minimum of 6 months but usually for many years, with features including fatigue, cognitive impairment, myalgias, post-exertional malaise, and immune system dysfunction. Dysregulation of cytokine signaling could give rise to many of these symptoms. Cytokines are present in both plasma and extracellular vesicles, but little investigation of EVs in ME/CFS has been reported. Therefore, we aimed to characterize the content of extracellular vesicles (EVs) isolated from plasma (including circulating cytokine/chemokine profiling) from individuals with ME/CFS and healthy controls.

**Methods:**

We included 35 ME/CFS patients and 35 controls matched for age, sex and BMI. EVs were enriched from plasma by using a polymer-based precipitation method and characterized by Nanoparticle Tracking Analysis (NTA), Transmission Electron Microscopy (TEM) and immunoblotting. A 45-plex immunoassay was used to determine cytokine levels in both plasma and isolated EVs from a subset of 19 patients and controls. Linear regression, principal component analysis and inter-cytokine correlations were analyzed.

**Results:**

ME/CFS individuals had significantly higher levels of EVs that ranged from 30 to 130 nm in size as compared to controls, but the mean size for total extracellular vesicles did not differ between groups. The enrichment of typical EV markers CD63, CD81, TSG101 and HSP70 was confirmed by Western blot analysis and the morphology assessed by TEM showed a homogeneous population of vesicles in both groups. Comparison of cytokine concentrations in plasma and isolated EVs of cases and controls yielded no significant differences. Cytokine-cytokine correlations in plasma revealed a significant higher number of interactions in ME/CFS cases along with 13 inverse correlations that were mainly driven by the Interferon gamma-induced protein 10 (IP-10), whereas in the plasma of controls, no inverse relationships were found across any of the cytokines. Network analysis in EVs from controls showed 2.5 times more significant inter-cytokine interactions than in the ME/CFS group, and both groups presented a unique negative association.

**Conclusions:**

Elevated levels of 30-130 nm EVs were found in plasma from ME/CFS patients and inter-cytokine correlations revealed unusual regulatory relationships among cytokines in the ME/CFS group that were different from the control group in both plasma and EVs. These disturbances in cytokine networks are further evidence of immune dysregulation in ME/CFS.

## Background

Myalgic Encephalomyelitis/Chronic Fatigue Syndrome (ME/CFS) is a life-limiting illness characterized by persistent debilitating fatigue, cognitive dysfunction often referred to as “brain fog”, pain/myalgias, post-exertional malaise, autonomic disturbances and gastrointestinal and immune system dysfunction lasting for at least 6 months, but usually much longer as prognosis for recovery is poor [1]. This symptomatology has led to the suspicion that ME/CFS is an inflammatory disorder; therefore, researchers have been investigating potential biomarkers including, oxidative stress [2, 3], energy metabolism [4, 5], or cytokine profiling (reviewed in [6–8]). Dysregulation of cytokine profiles has been demonstrated and often associated with an enhancement of pro-inflammatory cytokines in patients with ME/CFS [9–13]. However, results are frequently inconsistent between studies and it remains difficult to determine a specific cytokine signature that could be implicated in the etiopathogenesis of ME/CFS.

Cytokines are important modulators of immune function and inflammatory responses and are easily affected by several factors which could be responsible for the high heterogeneity observed among studies. They are generally considered to function as soluble factors that mediate cell-to-cell communications in multicellular organisms. Their secretion can occur in classical secretion manner or via extracellular vesicles (EVs).

EVs are heterogeneous membrane-enclosed structures, spherical in shape with different origins, size and composition and released by cells into the extracellular milieu. A wide variety of EV subtypes have been proposed, defined by their size, biogenesis pathway, cargo, cellular source, and biological function, leading to a historically heterogeneous nomenclature including terms like exosomes and ectosomes. They may be released from damaged or stressed cells and carry proteins, pathogen-associated and damage-associated molecular patterns, lipids and miRNA, which are encapsulated and thus protected from degrading enzymes [14]. Cytokines may be selectively sorted to EVs and act as signals to regulate and propagate the immune and inflammatory response [15]. EV-associated cytokines are different from those released in a soluble form by cells of the same type [16]. Once secreted, EVs can be rapidly captured by dendritic cells, phagocytes or macrophages [17], bind neighboring cells that express specific cytokine receptors, or circulate passively through the bloodstream to sites distant to the local inflammatory lesion. A recent study reported that EV-associated and encapsulated cytokines were more stable than free cytokines and became biologically active upon interacting with sensitive cells [16].

There is increased evidence that EVs are one of the main participants in cell-to-cell communication [18] and drive inflammatory, autoimmune and infectious disease pathology [19–22]. They can be sources of proinflammatory cytokines such as Tumor Necrosis Factor-α (TNF- α), Interleukins -6, -1β, -8 (IL-6, IL-1β, IL-8), Monocyte Chemoattractant Protein-1 (MCP-1), can stimulate their production in a variety of cells, and can also induce activation and proliferation of B and T cells as well as migration of granulocyte into inflamed tissues, promoting inflammatory pathways in recipient cells.

In previous reports, circulating EVs have been shown to be increased in number in ME/CFS [23–25], results in line with other diseases such as cancers, Alzheimer’s and Parkinson’s disease [21, 26–28]. Furthermore, a recent study was able to distinguish ME/CFS from idiopathic chronic fatigue and clinical depression by analyzing the content of EVs by means of proteomics [25]. Observations in that study were an upregulation of proteins involved in focal adhesion, regulation of the actin cytoskeletal signaling pathway and pathways relevant to Epstein-Barr virus infection. Another report showed that neuronal and endocrine system pathways were among differentially expressed miRNA in EVs in ME/CFS, which suggests that there are defects in endocrine tissue functioning [23].

Unlike our pilot study described below, the prior studies did not report on the cytokine composition of extracellular vesicles isolated from plasma of ME/CFS patients. We utilized an immune-profiling approach to determine whether an abnormal set of EV-associated cytokines could be identified in ME/CFS.

## Materials and methods

### Participants

Cases and controls were recruited by Susan Levine, M.D. (Manhattan, NY) between November 19, 2013 and October 21, 2014. A total of 35 ME/CFS cases and 35 healthy controls were included in this age- and sex-matched case–control cross-sectional study. Patients were diagnosed with ME/CFS if they met the 1994 CDC/Fukuda definition [29] and controls were eligible if they did not have history of fatigue and did not meet the ME/CFS case definition. Individuals with a known acute illness or chronic infectious disease were excluded from the study. A number of subjects were taking psychoactive medications and were not asked to stop them to be included in the study.

Non-fasting peripheral blood was drawn in EDTA tubes in the morning and was stored on the same day of collection at −80 °C until further processing. Participants’ age, sex, and age of onset of ME/CFS were recorded. The Bell’s disability scale [30], Short Form-36 Health Survey [31] and Symptom Severity Scale (SSS) were administered to each participant on the day of blood sample collection. A higher score on the SSS indicates greater severity. As indicated in Table 1, mean of ages and BMI of cases and controls were within 3 years and the female to male ratios were equal. Written consent was obtained from all participants and all protocols were approved by the Cornell University Institutional Review Board, approval # 1303003741.

**Table 1:** Study population characteristics

	ME/CFS	Controls	Mann–Whitney U test
Age (years)	50.6 ± 13.7	50.2 ± 17.5	*p *=* 0.42*
Gender			
Female	28	28	NA
Male	7	7	NA
BMI (kg/m^2^)	24.3 ± 5.3	24.3 ± 4.6	*p *=* 0.18*
Onset of disease			
Gradual	40%	NA	NA
Sudden	60%	NA	NA
Bell score	35.4 ± 14.1	96.3 ± 9.5	*p *<* 0.001*
SF-36			
Physical function	44.1 ± 17.2	95.9 ± 7.3	*p *<* 0.001*
Role physical	5.0 ± 11.8	91.4 ± 26.1	*p *<* 0.001*
Pain	40.8 ± 21.6	87.3 ± 14.1	*p *<* 0.001*
General health	25.9 ± 14.5	87.3 ± 15.9	*p *<* 0.001*
Vitality	17.9 ± 15.8	75.4 ± 17.8	*p *<* 0.001*
PCS^a^	26.8 ± 7.5	55.1 ± 5.2	*p *<* 0.001*
Social function	33.6 ± 22.6	94.8 ± 12.3	*p *<* 0.001*
Role emotional	45.7 ± 45.1	91.9 ± 24.6	*p *<* 0.001*
Mental health	59.3 ± 19.8	79.7 ± 18.6	*p *<* 0.001*
MCS^a^	38.9 ± 10.9	53.4 ± 9.6	*p *<* 0.001*
SSS^a^			
Fatigue	6.9 ± 2.5	1.4 ± 1.7	*p *<* 0.001*
Impaired memory	6.4 ± 2.8	0.8 ± 1.4	*p *<* 0.001*
Recurrent sore throat	3.9 ± 3.3	0.4 ± 1.1	*p *<* 0.001*
Tender lymph nodes	3.9 ± 3.1	0.1 ± 0.4	*p *<* 0.001*
Muscle tenderness or pain	5.5 ± 2.9	1.5 ± 1.9	*p *<* 0.001*
Joint pain	5.1 ± 2.9	1.5 ± 2.0	*p *<* 0.001*
Headache	5.0 ± 3.4	0.7 ± 1.4	*p *<* 0.001*
Disturbed sleep or waking unrefreshed	7.6 ± 3.0	1.6 ± 2.1	*p *<* 0.001*
Post-exertional malaise	7.8 ± 2.7	0.8 ± 1.7	*p *<* 0.001*

^a^*PCS* Physical component score, *MCS* Mental component score, *SSS* Specific symptom severity (0 = none, 10 = very severe)

### Extracellular vesicle isolation and characterization

Total extracellular vesicles (EVs) were isolated from 750 μl of plasma by precipitation using the ExoQuick™ reagent (System Biosciences, Palo Alto, CA, USA). Plasma samples from each subject were thawed on ice and centrifuged at 3000×*g* for 15 min at room temperature to remove cells and debris. The supernatant was transferred to a new tube, and thrombin (611 U/ml) (System Bioscience, Palo Alto, CA, USA) was added and samples were incubated for 5 min at room temperature to remove fibrinogen, centrifuged at 10,000×*g* for 5 min, and the supernatant was collected. The samples were then incubated with ExoQuick™ for 60 min at 4 °C. The ExoQuick™/serum-like samples were then centrifuged at 12,000×*g* for 5 min, and the resulting pellet was resuspended in 250 ul of sterile phosphate buffered saline 1X, pH 7.4. To prevent aggregation and cryodamage, 25 mM of trehalose was added to the isolated EV fraction [32]. Samples were aliquoted for total protein determination, Western blot analysis, Nanoparticle Tracking Analysis (NTA), Transmission Electron Microscopy (TEM), and measurement (quantification) of cytokines/chemokines and growth factors.

### Protein quantification and western blot analysis

Total protein quantification from EV isolates was performed using the Pierce^TM^ BCA Protein Assay kit (ThermoFisher Scientific) according to manufacturer’s instructions. The assay is a detergent-compatible formulation based on bicinchoninic acid for the colorimetric detection (A562 nm) and quantitation of total protein.

Purified EVs were assayed for Western blot analysis (WB). WB is used to validate the presence or absence of EV protein markers in purified samples based on the availability of specific antibodies CD63, CD81, HSP70 and TSG101 (System Biosciences, LLC, Palo Alto, CA, USA). Protein samples were prepared by adding 100 μl ice-cold RIPA buffer containing protease/phosphatase inhibitors to 100 μl extracted EV samples resuspended in the appropriate buffer. EV lysates were adjusted to the same protein content (150 μg), denatured for 10 min in 2X Laemmli buffer, resolved by 8–10% SDS-PAGE, and then proteins were transferred to PVDF membranes (Amersham, GE Healthcare, USA). Membranes were blocked in 5% non-fat dry milk using TBS containing 0.1% Tween-20 and then incubated with various primary polyclonal antibodies (anti-CD63, anti-CD81, anti-HSP70, anti-TSG101; dilution 1/500; Santa Cruz Biotech, CA, USA) and cytochrome C antibody as a negative control for overnight at 4 °C, washed with 1X TBS-T and then incubated with secondary conjugated antibody for 1 h at room temperature. X-ray films were exposed in a darkroom and films were developed and visualized using and infrared Odyssey machine (LI-COR Biosciences).

### Nanoparticle tracking analysis

Extracellular vesicles’ concentration and size distribution were assayed in samples using a NanoSight NS300 (Malvern). Samples were thawed and diluted to 1:2000 in PBS 1X and 1 ml was injected through the laser chamber (NanoSight Technology, London, UK). The NanoSight NS300 uses a source light to illuminate nanoscale particles (30–800 nm) as point scatters moving under Brownian motion. Three recordings of 60-second digital videos of each sample were acquired and analyzed by the NanoSight NTA 2.3 software to determine the size and the concentration of nanoparticles. Results were averaged together.

### Transmission electron microscopy

EV suspensions were visualized under a 120 kV field emission transmission electron microscope (FEI T12 Spirit TEM/STEM) at the Cornell Center for Materials Research in Ithaca, NY. Isolated EV suspensions were thawed and diluted at either 1:100, 1:500, 1:1000 or 1:2000 in 2% PFA overnight at 4 °C before proceeding with negative staining. Samples were applied to copper 300-mesh Formvar coated carbon stabilized grids and were allowed to adsorb to the grid for 20 min. Grids were then washed in PBS 1X and transferred to a 1% glutaraldehyde solution for one min post-fixation. Samples were then washed 8 times by floating on distilled water for 2 min. Negative staining was then achieved through placing the grids on a drop of 2% Aqueous Uranyl Acetate for 10 min followed by air drying and storage in an EM grid box.

### Cytokine, chemokine and growth factor measurement in EV and plasma samples

Due to cost considerations for cytokine assays, a subset of 19 females with ME/CFS and 19 healthy females out of the 70 initial samples, age and BMI-matched, were used. Both purified EVs and plasma from every subject were analyzed for cytokines, chemokines and growth factors using a human 45-plex magnetic bead kit (R&D Systems, Minneapolis, USA). EV samples were treated with Triton 1% to allow the release of encapsulated cytokines [16]. Each sample was measured in duplicate on a MAGPIX^®^ Multiplexing System (Luminex Corp.) at the Human Nutritional Chemistry Service Laboratory at Cornell University. For each well, we used the median fluorescence intensity (MFI) of all beads measured for a given analyte and averaged the MFI of the two replicates and results were accepted when the coefficient of variation (CV) was below 15%. MFIs for analytes are best for analysis because fluorescence enables the analysis of low signals and have power for testing differences in analyte expression [33–35].

### Statistical analysis

All data was processed and analyzed using R version 3.5.1 (2018-07-02) via RStudio Version 1.2.5033. The independent samples *t* test and non-parametric Wilcoxon-Mann–Whitney U test were used to determine the significance of differences (p < 0.05) in each subject group for age, BMI, EV size and concentrations. A linear model analysis was used to compare cytokine measurements from plasma and EVs between groups with age and BMI as confounding variables. Principal Component Analysis (PCA) was used to sum up and to simplify the data by reducing the dimensionality of the cytokine levels datasets. Variables were first “mean-centered” and scaled to have standard deviation one. Then, the data correlation matrix was calculated and eigenvalue decomposition on the matrix was performed. Cluster tendency of the datasets was assessed using the Hopkins statistic (H) [36] by measuring the probability that a given data set is generated by a uniform data distribution. A H value close to 1 tends to indicate the data is highly clustered, and random data will tend to result in values close or below 0.5. PCA and cluster tendency were performed using the R packages *FactoMineR*, *factoextr* and *clustertend*.

Spearman’s rank correlation coefficients were also estimated between each cytokine and the metadata (age, BMI, Bell, SF-36 and Symptom Severity Scale scores) and partial Spearman’s rank correlation were performed adjusting for age and BMI. Post hoc tests were run to assess the differences between ME/CFS and controls using the R *emmeans* package. Throughout, all p-values were corrected using a Benjamini and Yekutieli correction to control the false discovery rate (FDR) under dependency of the cytokines with a 0.05 family-wise False Discovery Rate [37]. The relationships among the analytes were analyzed within each group allowing the discovery of different cytokine-cytokine structures across the different group populations. Significantly correlated cytokine pairs were displayed on a network diagram using *igraph*, *limma* and *ggplot2* R packages, in which cytokines are represented by nodes and significantly correlated cytokines are connected by edges.

## Results

### Cohort characteristics

Within the study population, there were 28 females and 7 males in both the ME/CFS and healthy controls (HC) groups (Table 1). All patients who were selected met the 1994 Fukuda definition [29] for ME/CFS. The average age was similar between both groups at 50.2 ± 17.5 in controls and 50.6 ± 13.7 in patients (p = 0.42, Table 1). Average Body Mass Index (BMI) was nearly identical at 24.3 ± 4.6 in controls and 24.3 ± 5.3 in patients (p = 0.18, Table 1). Sixty percent of the ME/CFS patients were able to identify an acute, often flu-like, illness that immediately preceded the onset of the disease, while 40% are unaware of an initiating event and consider their onset to be gradual (Table 1), and all patients had the illness for more than 10 years. Bell Scale ratings were significantly different between groups, with scores averaging 35.4 ± 14.1 and 96.3 ± 9.5 for ME/CFS and controls, respectively (p < 0.001, Table 1). Additionally, both the Physical and Mental Component Scores (PCS and MCS respectively) derived from the SF-36 short survey were, as expected, higher in the control group (p < 0.001, Table 1). All symptoms from the Specific Severity Symptom scale were higher in the ME/CFS group (Table 1).

### Characterization of extracellular vesicles

**Fig. 1:**
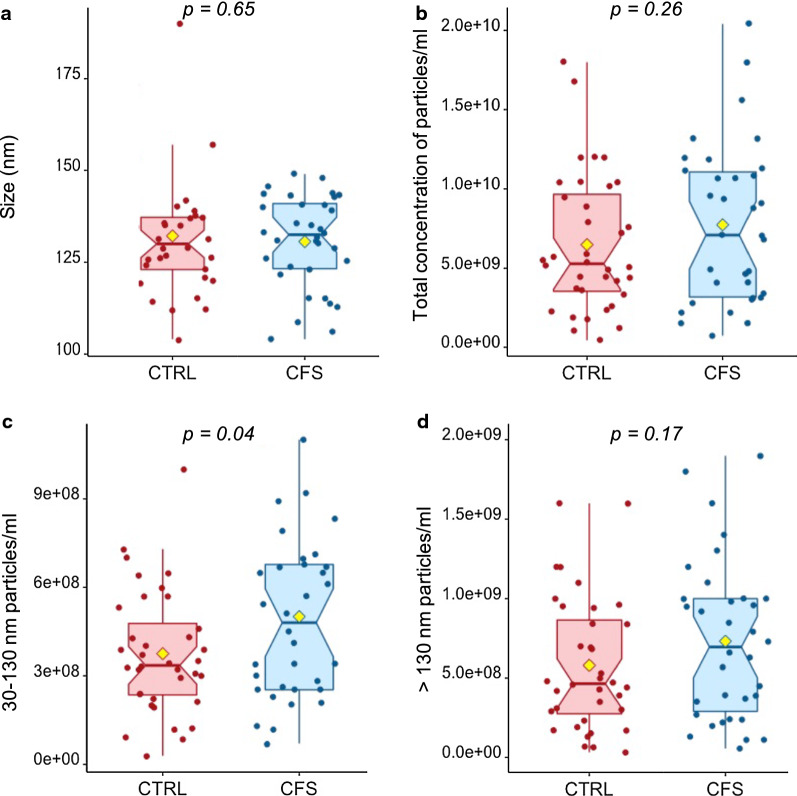
Sizing and quantification of Extracellular Vesicles. Size in nm (**a**), total concentration (**b**), 30–130 nm concentration (**c**) and > 130 nm concentration (**d**) of particles per ml of plasma in ME/CFS subjects and healthy controls (CTRL) as determined by Nanoparticle Tracking Analysis. The yellow dot represents the mean

We investigated whether there were differences in levels and size of circulating extracellular vesicles purified from plasma samples from ME/CFS patients and healthy individuals. EVs were purified by precipitation using ExoQuick^TM^ reagent and then analyzed by Nanoparticle Tracking Analysis (NTA), Transmission Electron Microscopy (TEM) and Western blotting. Protein levels of total EV fractions were measured by BCA (bicinchoninic acid assay) and no significant difference was found between ME/CFS and controls (21.9 mg/ml ± 1.1 and 23.2 mg/ml ± 0.9, respectively) (p = 0.296). Sizing and quantification of EVs were performed using a NanoSight NS300 instrument on all samples (N = 35 for both ME/CFS and control samples) and results are shown in Fig. 1. Accumulating data have indicated that the EVs are highly heterogeneous and classically defined by their size and content. At present, at least 3 main subgroups of EVs have been defined: apoptotic bodies (1– 5 µm), large microvesicles (130-1000 nm), and small EVs (exosomal) (30–130 nm) [38, 39]. All nanoparticles purified were smaller than 500 nm, most of them being in the typical exosome size range of 30–130 nm. Nanoparticle Tracking Analysis revealed that EV particles’ size means did not differ between healthy individuals (132.7 ± 16.4 nm, range 112–190 nm) and ME/CFS patients (130.1 ± 12.7 nm, range 114–149 nm) (p = 0.65, Fig. 1a).

Although the mean total concentration of particles/ml of plasma (controls: 6.6 ± 4.3 x10^9^, ME/CFS: 7.6 ± 5.0 x 109; p = 0.26, Fig. 1b) and the mean concentration of particles greater than 130 nm (controls: 5.0 ± 4.2 x10^8^; ME/CFS: 7.1 ± 5.0 x 10^**8**^, p = 0.17, Fig. 1d) did not exhibit a statistically significant difference between groups, the mean concentration of EVs that ranged from 30 to 130 nm in size was statistically different (3.8 ± 2.1 x 10^8^ and 4.9 ± 2.8 x10^8^ particles/mL of plasma for healthy controls and ME/CFS subjects, respectively) (p = 0.04, Fig. 1c).

**Fig. 2:**
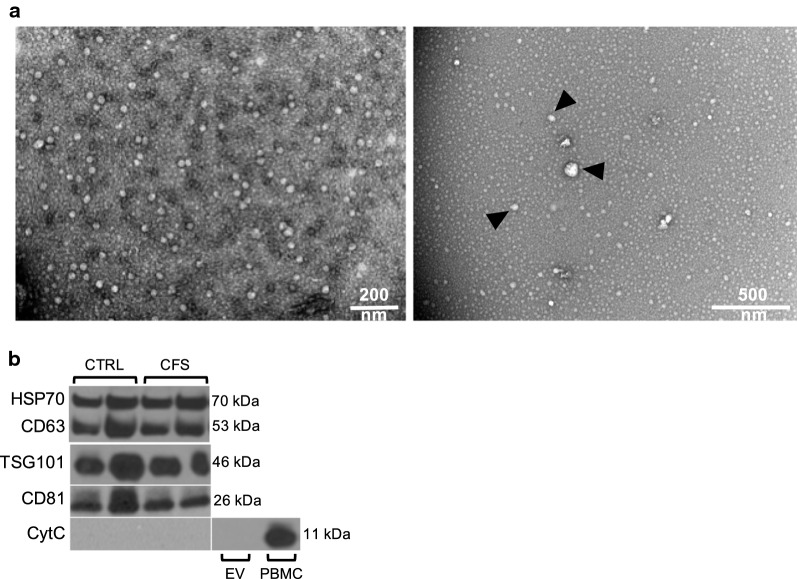
Characterization of Extracellular Vesicles. (**a**) Morphology of isolated EVs from ME/CFS was confirmed by transmission electron microscopy (**a** representative image is shown; scale bars: 200 nm and 500 nm) and (**b**) Western blot analysis of isolated EVs from healthy controls (CTRL) and ME/CFS subjects as representative samples. Thirty μg of protein was loaded in each lane and probed with specific antibodies to EV protein markers. Cytochrome C (mitochondrial marker) was used as negative control for EV and positive for PBMCs

Representative EV isolates from both groups were further analyzed by transmission electron microscopy (TEM) on 300-mesh copper Formvar-coated grids. EV isolates were clearly visualized and morphological analysis revealed a homogeneous population of vesicles that were spherical-shaped, enclosed by a lipid bilayer, and had a size distribution that agrees with the NTA data (Fig. 2a).

To further confirm the presence of EVs in the samples, we investigated EV fractions of 10 subjects from each group by Western blotting. The enrichment of typical EV markers was assessed using specific antibodies for the tetraspanin family proteins CD63 and CD81, the ESCRT-associated protein Tumor Susceptibility Gene 101 (TSG101), and the cytosolic heat shock protein HSP70. Two representatives of each group are shown in Fig. 2b. The analysis confirmed that CD63-, CD81-, TSG101- and HSP70-positive nanoparticle populations were recovered. The mitochondrial protein cytochrome C was used as a negative control and its absence in EV isolates from both ME/CFS patients and healthy controls demonstrate that the samples were free of cellular protein contamination (Fig. 2b).

### Cytokine content in plasma and extracellular vesicles

A subset of 38 samples out of the 70 analyzed previously (19 ME/CFS females and 19 females controls) were subjected to measurement of 45 cytokines/chemokines and growth factors levels through a multiplex assay on both plasma and purified EV samples and their levels were compared using mean MFI (Table 2). Of the 45 analytes measured, cases and controls were not distinguished overall as there were no significant differences between the groups after correction for multiple comparisons. Even though Interleukin-17E (IL-17E) levels in EVs and MCP-1 levels in both plasma and EVs (Table 2) initially appeared significantly different, they did not remain significant after FDR correction (Table 2). Cytokine level ratios (ME/CFS vs. controls) showed that the majority of the cytokines were at similar levels in both groups and type of samples with the exception of a few that were increased or decreased in the ME/CFS group (Table 2).

**Table 2:** Comparison of plasma immune analytes in plasma and EVs from individuals with ME/CFS and healthy controls

Plasma				Extracellular vesicles			
Cytokine	P-value	Q-value	CFS/controls	Cytokine	P-value	Q-value	CFS/controls
CD40 ligand	0.422	0.863	1.0	CD40 ligand	0.421	0.858	1.0
EGF	0.122	0.863	0.7	EGF	0.331	0.858	0.8
Eotaxin	0.931	0.983	0.9	Eotaxin	0.301	0.858	1.1
FGF-β	1.000	1.000	1.0	FGF-β	0.553	0.858	1.0
Flt3 ligand	0.350	0.863	1.1	Flt3 ligand	0.784	0.909	1.0
Fractalkine	0.118	0.863	1.1	Fractalkine	0.584	0.858	1.0
G-CSF	0.396	0.863	1.0	G-CSF	0.503	0.858	1.0
GM-CSF	1.000	1.000	1.0	GM-CSF	0.089	0.858	1.4
Granzyme B	0.683	0.940	1.0	Granzyme B	0.855	0.933	1.0
Groα	0.773	0.940	1.0	Groα	0.796	0.909	1.1
Groβ	0.313	0.863	0.8	Groβ	0.641	0.858	0.5
IFN-α	0.521	0.937	0.9	IFN-α	0.456	0.858	0.9
IFN-β	0.661	0.940	0.9	IFN-β	0.952	0.952	1.5
IFN-γ	0.737	0.940	1.0	IFN-γ	0.727	0.909	1.1
IL-12p70	0.255	0.863	0.9	IL-12p70	0.104	0.858	1.9
IL-13	0.413	0.863	0.8	IL-13	0.563	0.858	1.0
IL-15	0.726	0.940	1.0	IL-15	0.627	0.858	1.7
IL-17	0.815	0.940	1.0	IL-17	0.386	0.858	1.3
IL-17E	0.630	0.940	0.9	IL-17E	*0.049*	0.858	0.6
IL-1RA	0.053	0.863	1.2	IL-1RA	0.346	0.858	0.9
IL-1α	0.115	0.863	0.9	IL-1α	0.952	0.952	0.9
IL-1β	0.661	0.940	1.0	IL-1β	0.465	0.858	0.9
IL-2	0.266	0.863	0.9	IL-2	0.202	0.858	1.6
IL-3	0.306	0.863	1.0	IL-3	0.796	0.909	1.1
IL-33	0.380	0.863	1.0	IL-33	0.236	0.858	1.2
IL-4	0.793	0.940	0.9	IL-4	0.616	0.858	0.9
IL-5	0.473	0.925	1.0	IL-5	0.594	0.858	1.1
IL-6	0.903	0.983	1.0	IL-6	0.648	0.858	1.1
IL-7	0.299	0.863	1.0	IL-7	0.378	0.858	1.8
IL-8	0.405	0.863	0.7	IL-8	0.808	0.909	1.2
IL-10	0.803	0.940	1.0	IL-10	0.784	0.909	1.3
IP-10	0.249	0.863	1.4	IP-10	0.162	0.858	1.5
MCP-1	*0.008*	0.338	1.5	MCP-1	*0.022*	0.858	1.7
MIP-1α	0.414	0.863	1.3	MIP-1α	0.475	0.858	1.3
MIP-1β	0.939	0.983	1.0	MIP-1β	0.879	0.933	0.9
MIP-3β	0.599	0.940	1.1	MIP-3β	0.891	0.933	1.2
MIP-3α	0.213	0.863	0.9	MIP-3α	0.485	0.858	0.9
PD-L1	0.726	0.940	1.0	PD-L1	0.634	0.858	1.1
PDGF-AA	0.354	0.863	0.9	PDGF-AA	0.425	0.858	0.8
PDGF-BB	0.181	0.863	0.8	PDGF-BB	0.086	0.858	0.6
RANTES	0.116	0.863	0.7	RANTES	0.221	0.858	0.7
TGF-α	0.521	0.937	1.0	TGF-α	0.504	0.858	1.1
TNF-α	0.748	0.940	1.0	TNF-α	0.466	0.858	1.2
TRAIL	0.838	0.942	1.0	TRAIL	0.218	0.858	1.3
VEGF-A	0.683	0.940	1.0	VEGF-A	0.230	0.858	1.1

p-values are shown prior correction for multiple comparison using the Benjamini and Yekutieli control for false discovery rate (q-values) at a 5% rate. Results were adjusted for age and BMI. Also indicated, are ratios of analyte levels ME/CFS vs. controls. Italics values denote statistical significance at p < 0.05

When cytokine levels in EVs were normalized with either the total protein concentration measured by BCA, the total concentration of particles/ml or the concentration of 30-130 nm particles/ml of plasma measured with NTA, there were no significant differences between groups (*data not shown*).

**Fig. 3:**
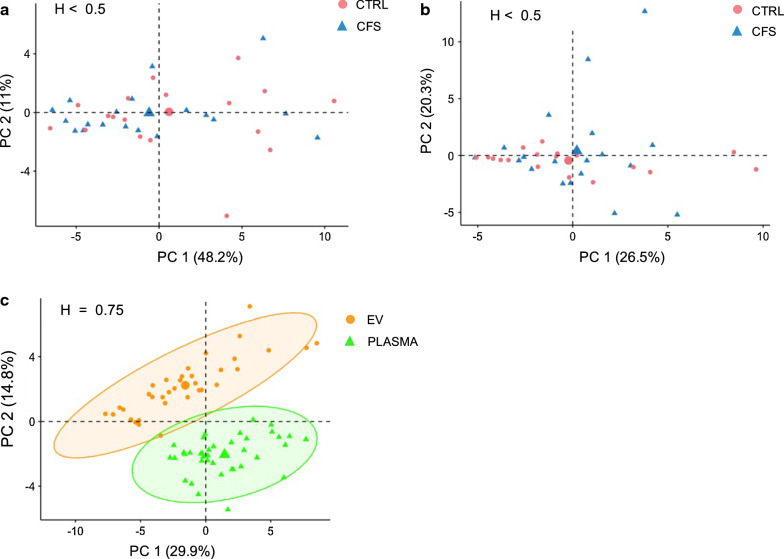
Principal Component analysis of cytokine levels in ME/CFS vs. controls. In plasma (**a**), in EVs (**b**) and (**c**) cytokine levels in EVs vs. plasma. The Hopkins statistic values H are shown for each PCA

Principal Component Analysis performed on cytokine levels explained 59.3% of the data distribution in plasma samples (PC-1 48.2%; PC-2 11%, Fig. 3a) and 46.8% in EV samples (PC-1 26.5%; PC-2 20.3%, Fig. 3b). Within plasma and EV samples, there appears to be no clear difference in between the ME/CFS group and healthy group (H < 0.5, Fig. 3a, b). Comparing sample types within subjects, the percentage of variability explained by each dimension was 29.9% for the first axis and 14.8% for the second axis and two significant clusters were observed, supported by the Hopkins statistic H = 0.75 (Fig. 3c).

Partial and Spearman ranked correlation analyses were performed between cytokine levels measured in plasma and EVs and other variables such as age, BMI, Bell Scale, the SF-36 scores, the Specific Severity Symptom scale scores and EV characteristics. In ME/CFS plasma samples, we found 3 significant positive correlations that were not found in the plasma from controls. IL-1RA positively correlated with age (r = 0.67, p = 0.002, Additional file 1: Figure S1a) and BMI (r = 0.71, p = 0.0006, Additional file 1: Figure S1b). The basic fibroblast factor FGF-β was strongly correlated with fatigue (r = 0.58, p = 0.009, Additional file 1: Figure S1c) before and after controlling for potentially confounding variables age and BMI.

Additionally, we also observed a number of significant correlations between EV cytokines and other variables that were unique to a group. Interestingly, the Macrophage inflammatory proteins MIP-1β, MIP-3β and Interleukin-2 (IL-2) had significant positive correlations with the concentration of the 30-130 nm population of particles in the control EV samples (r = 0.61, p = 0.008 for MIP-1β, r = 0.67, p = 0.002 for MIP-3β and r = 0.6, p = 0.009 for IL-2, Additional file 2: Figure 2a, b, c) that were not present in the EV samples from patients. Transforming Growth Factor α (TGFα) and Interleukin-33 were found to inversely correlate with headaches in the ME/CFS EV samples (r = −0.66, p = 0.002 and r = −0.63, p = 0.005 for TGFα and IL-33 respectively, Additional file 2: Figure 2d, e), and these correlations were unique in patients with ME/CFS compared to the control group.

**Fig. 4:**
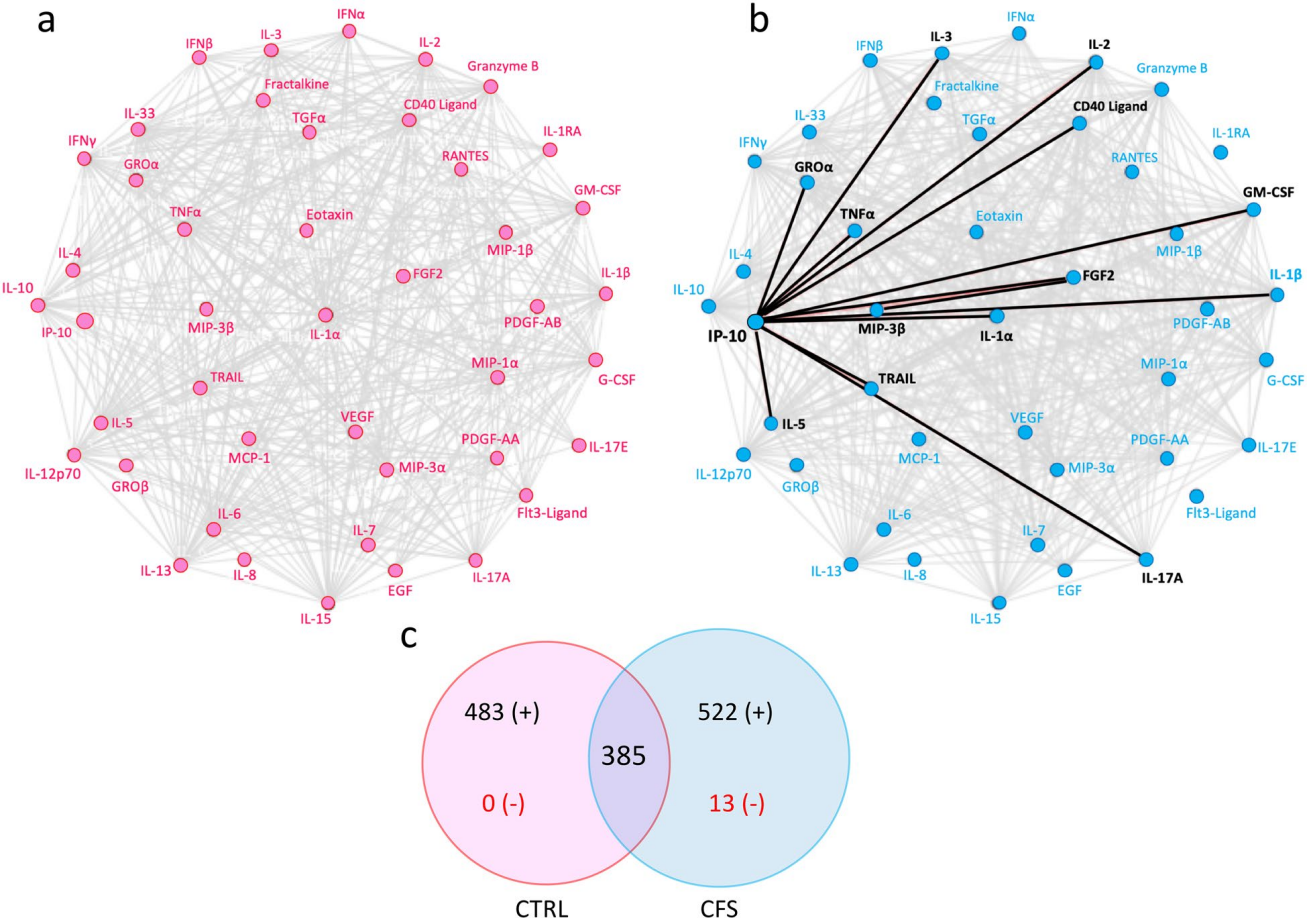
Cytokine–cytokine correlations in plasma. Network diagrams of cytokine interactions in plasma of ME/CFS patients (**a**), and in controls (**b**). Grey edges indicate positive correlations and black bold lines negative associations. The Venn diagram (**c**) shows the number of significant cytokine-cytokine associations common to both groups and unique to each group. In red are the number of negative correlations. All correlations in this analysis were determined using partial Spearman’s ranked correlation adjusted for age and BMI

**Fig. 5:**
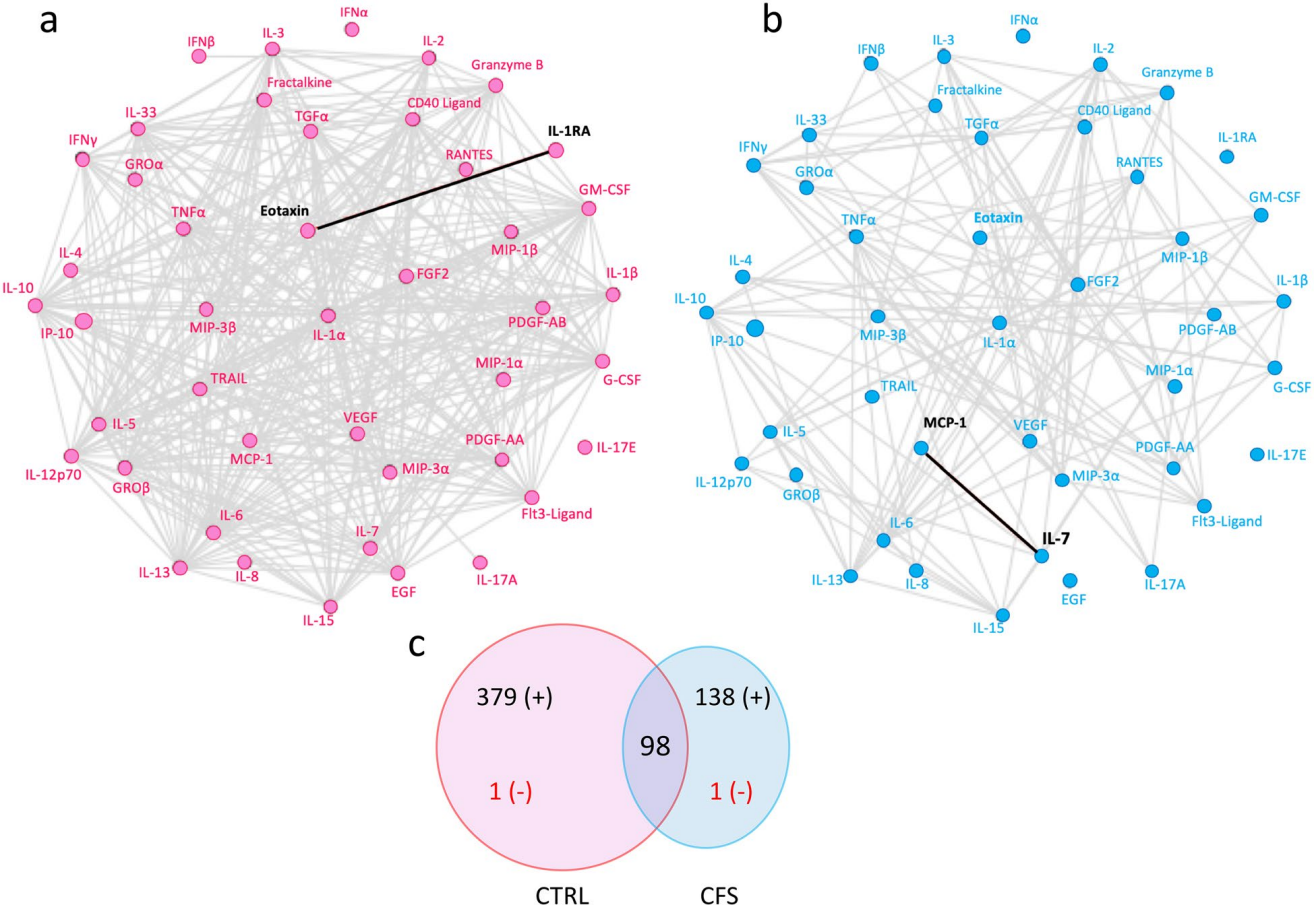
Cytokine–cytokine correlations in extracellular vesicles. Network diagrams of cytokine interactions in EVs of ME/CFS patients (**a**), and in controls (**b**). Grey edges indicate positive correlations and black bold lines negative associations. The Venn diagram (**c**) shows the number of significant cytokine-cytokine associations common to both groups and unique to each group. In red are the number of negative correlations. All correlations in this analysis were determined using partial Spearman’s ranked correlation adjusted for age and BMI

Next, we investigated cytokine-cytokine interactions in plasma and EV samples from both groups. These analyses were intended to determine whether these analytes revealed specific regulatory relationships. Network diagrams showed cytokine-cytokine correlations patterns that differed between ME/CFS and controls in plasma as well as in EV samples (Figs. 4  and 5).

A total of 385 significant cytokine-cytokine interactions were common to both groups in plasma samples but a higher significant number of interactions was present solely in plasma from ME/CFS patients in comparison to the controls (535 and 483 respectively, Fig. 4c). Whereas in the plasma of controls no inverse relationships were found across any of the cytokines (Fig. 4a and c), a total of 12 such associations (Fig. 4b, c) were found in the plasma of case subjects with IP-10 (IP-10/GROα; IP-10/IL-3; IP-10/TNFα; IP-10/IL-2; IP-10/CD40 Ligand; IP-10/GM-CSF; IP-10/FGF-β (FGF2); IP-10/IL1-α; IP-10/IL-1β; IP-10/TRAIL; IP-10/IL-5 and IP-10/IL-17A) and MIP-3β with FGF2.

In EVs from controls there were > 2.5 more significant inter-cytokine correlations than in the ME/CFS group (380 vs. 139 respectively, Fig. 5c) and the two groups shared a total of 98 significant inter-cytokine correlations. Interestingly, we found one negative correlation in each network that was different: IL-RA inversely correlated with Eotaxin in the control group (Fig. 5a) while MCP-1 was negatively correlated with IL-7 in the ME/CFS group (Fig. 5b).

## Discussion

Extracellular vesicles were isolated from the plasma of 35 patients with ME/CFS and 35 healthy controls. Transmission electronic microscopy and Western blot analyses on isolated EVs revealed mainly an exosomal morphology with mean sizes ranging from 30 to 130 nm along with the detection of exosomal markers (CD63, CD81, HSP70 and TSG101) (Fig. 2). Contrary to two previous reports that found that the mean size of ME/CFS EVs was reduced [23, 24], we did not find any statistical significant difference in our study population. Although the total concentration of particles per ml of plasma did not differ between patients and controls in our study, the concentration of 30-100 nm vesicles was significantly increased in the diseased group (Fig. 1c), confirming findings recently reported for ME/CFS [23–25]. These results are also in line with other conditions such as breast and gastric cancer [26, 28], Alzheimer’s disease [21] or cerebrovascular disease [27] in which EVs were found to be elevated in the disease state. Oxidative stress, changes in intracellular calcium level and synaptic activity are contributing factors for inducing exosome secretion [40, 41].

We further investigated the cytokine content in EVs and plasma from a subset of 19 ME/CFS and 19 healthy females. Using a linear model regression analysis that adjusted for age and BMI on the 45 cytokines measured, we did not find any statistically significant differences between groups in the plasma or EV cytokine levels. Furthermore, the data distribution in both plasma and EVs assessed by PCA did not show any clustering that could distinguish ME/CFS patients (Fig. 3a, b). Within subjects, the cytokine levels in EVs and plasma appeared as two different clusters (Fig. 3c) confirming that EV and plasma are two different compartments.

In a larger cohort analyzed by Hornig et al. [10], nine plasma cytokine levels significantly differed between cases and controls. Montoya et al. [12] found only two cytokines out of 51 measured to be significantly different in plasma of ME/CFS patients when compared with healthy individuals (Resistin was lower and TGF-β was elevated). Considering the limited number of analyzed samples in our pilot study (ME/CFS, n = 19 and controls, n = 19) in comparison to Hornig et al. (ME/CFS, n = 298 and controls, n = 348) or Montoya et al. (ME/CFS, n = 186 and controls, n = 38), it is not surprising that we did not find any differences between groups, as a result of low statistical power.

When comparing short duration (≤ 3 years) to long duration (> 3 years) of illness, several studies reported differences in plasma cytokine levels between ME/CFS subgroups and healthy individuals. Landi and colleagues [42] compared the plasma cytokine levels of 100 ME/CFS of long duration to 79 controls and found reductions in 3 out of 31 cytokines measured (IL-7, IL-16, and Vascular Endothelial Growth Factor A, VEGF-A) while Hornig et al. observed that early ME/CFS cases (duration ≤ 3 years) showed statistically significant differences in comparison to controls for more than half of the 51 cytokines analyzed [10]. These findings were not reproduced by Montoya et al., who did not find any cytokine to be significantly different between short duration cases (≤ 3 years) or long duration cases (> 3 years) and healthy individuals [12]. Unfortunately, our analysis is limited as ME/CFS cases in this study were ill for more than 3 years, which prevented us from analyzing cytokine levels between short and long duration of illness.

Despite a lack of significance, higher and lower levels of several cytokines were found in plasma and EV samples as shown by the ratio of cytokine levels of ME/CFS vs. controls (Table 2). In both EVs and plasma samples, MIP-1alpha and IP-10 were both elevated in the ME/CFS group but not statistically different, a trend previously observed in a report that considered disease severity and compared mild ME/CFS patients to controls [12] but opposite to another study showing significant lower levels of these cytokines in ME/CFS plasma samples [10]. Interestingly, in our study, the Monocyte Chemoattractant Protein-1 (MCP-1 or CCL2) was significantly higher in both sample types in the ME/CFS group but lost statistical significance after correction for multiple comparisons (Table 2). MCP-1 is of relevance within the central nervous system (CNS), as it is expressed in different parts of the brain by neurons and microglia [43–45]. MCP-1 acts mainly as an attractor for mononuclear cells, a mediator of inflammation, but is also involved in neuroprotection against excitotoxic injuries [46, 47]. In ME/CFS, decreased levels of MCP-1 were observed in Cerebrospinal Spinal Fluid (CSF) samples in comparison to controls and these levels were higher in comparison to Multiple Sclerosis’ CSF samples [48]. Also, higher levels were reported in plasma obtained from ME/CFS patients with short duration of illness versus controls [10]. Elevated levels of MCP-1 have been also observed in several CNS-related pathologies and neurodegenerative diseases such as in the plasma of patients with Alzheimer’s disease [49, 50], amyotrophic lateral sclerosis (ALS) [51], in the cerebrospinal fluid of patients with ischemic stroke [52], in peripheral blood mononuclear cells obtained from Parkinson’s disease and ALS patients [51, 53], and in HIV-associated dementia [54], schizophrenia [55] and epilepsy [56]. Furthermore, correlations between concentration of MCP-1 and symptoms such as depression, anxiety and fatigue [57] or severity of disease have been reported [57, 58]. Due to its role in the development of the inflammatory and immune responses, MCP-1 could be considered as an indicator that might allow the detection and quantification of the progression of ME/CFS.

Several EV-associated cytokine levels appeared to be elevated in the ME/CFS group (Table 2) but were not statistically different probably due to our limited sample size. Amongst them, IL-7 which was previously found to be elevated in serum samples of severely afflicted ME/CFS patients [59], is a hematopoietic cytokine with critical functions in both B- and T-lymphocyte development. It is secreted by stromal cells, dendritic cells as well as neurons and neuronal progenitor cells [60] and previous studies have revealed that IL-7 promotes neuronal differentiation [61]. It can also stimulate cytotoxic functioning in mature T cells and NK cell proliferation, activities reduced in ME/CFS [62–64]. Hardcastle et al. [59] found a positive correlation between Interferon-γ (IFN-γ) and IL-7 in severely affected ME/CFS patients. Higher levels of IFNγ are associated with reduced Natural Killer cell cytotoxic activity observed in this illness and therefore, elevated levels of both cytokines may be involved in disease severity. In turn, lower IL-7 has been correlated with cognitive decline during aging [65] and a reduction in IL-7 in plasma previously observed in ME/CFS [42] suggests a reduction in immune activation along with a potential neuropathology similar to the process of aging.

Another example in our study, although not significant, were the elevated levels of the anti-inflammatory cytokine IL-10 in EVs isolated from ME/CFS patients. In contrast, extracellular vesicles levels of IL-10 levels were found to be reduced in gastric cancer [26]. IL-10 receptors are found on different populations of brain cells including astrocytes, oligodendrocytes, and microglia [66, 67]. The stimulation of these receptors with IL-10 reduces synthesis of proinflammatory cytokines, allowing the survival of brain cells [68–70]. Its expression is elevated during the course of several CNS-related diseases such as multiple sclerosis, Alzheimer’s disease [71] or meningoencephalitis [72] and promotes survival of neurons and all glial cells. In ME/CFS, studies showed contradictory results about levels of IL-10, but none measured it in extracellular vesicles. A longitudinal study reported increased blood IL-10 levels in patients [73], while others reported decreased [9, 74] and increased [75] levels in cerebrospinal fluid or plasma [10]. These findings are inconsistent and this may be due to the heterogeneity of the disease, different analytical methods, and the presence of different patient subgroups. Compromises to brain cells may contribute to low levels of IL-10, and a decrease in CNS IL-10 may be related to symptoms reported in ME/CFS cases. The role of IL-10 in ME/CFS requires further investigation. The elevated levels observed in this pilot are consistent with an immune activation.

While the levels of IL-2 in our plasma samples did not differ between groups, they were elevated in the ME/CFS EVs in comparison to controls (Table 2) but not statistically significant. IL-2 levels have been previously reported to be higher in CSF [48] and plasma from ME/CFS patients [76]. We can only speculate that these elevated cytokine levels found in EVs from our cohort of ME/CFS patients are part of a specific immune response in ME/CFS.

The content of different types of EVs reflect that of the parent cells and are enriched in certain molecules, including cytokines, chemokines, functional microRNAs, and cell-specific antigens. EVs maintain characteristics of the antigen presenting cell from which they are derived, exposing antigen‐presenting MHC I and MHC II molecules on their surface [77].

We further investigated if EV or plasma cytokine levels correlated with age, BMI and scores of the Bell, SF-36 and SSS forms, by performing Spearman’s and partial Spearman’s rank correlation adjusting for age and BMI. Sex was not included as a confounding variable in the analysis as all our participants were females. Even though several cytokines correlated either positively or inversely (Additional files 1 and 2: Figs. 1, 2) there were no commonalities between EV and plasma samples. IL-1RA was significantly and positively correlated with age and BMI in plasma samples from the ME/CFS group. It is well documented that IL-1RA, a natural antagonist to the proinflammatory cytokine IL-1, increases dramatically in obese subjects [78]. Furthermore, it has been shown that IL-1RA levels were positively correlated with serum leptin levels in ME/CFS [13] and that leptin was associated with fatigue severity in patients with ME/CFS [12, 13] but also with chronic hepatitis C and irritable bowel syndrome [79, 80]. Leptin has been identified as a major proinflammatory cytokine that induces IL-1RA secretion [81] and is involved in NK cell activation, and innate and acquired immune responses [82]. Unfortunately, our cytokine panel did not measure leptin and because of the close relationship existing between IL-1RA and leptin, we can only speculate that the higher levels of IL-1RA observed in ME/CFS plasma (Table 2) and the significant positive correlations of IL-1RA observed in the ME/CFS group and not in the control group may be due to increased inflammation in patients.

Interestingly, in EV samples from the control group, 3 cytokines strongly positively correlated with the 30-130 nm extracellular vesicle population (MIP-1β, MIP-3β and IL-2, Additional file 2: Fig. 2a–c) but not in the ME/CFS group. MIPs are crucial for immune responses towards infection and inflammation [83] and are produced by macrophages and monocytes, and stimulated by proinflammatory cytokines such as IL-1β [84]. IL-2 is a potent stimulator of T-cell proliferation and inhibits the development of inflammatory Th17 cells [85]. The fact that these cytokines correlated with a particular population of EVs in the control group suggest that ME/CFS patients may be lacking specific vesicles carrying important proteins able to respond to inflammatory challenges. Cytokines and chemokines can be selectively sorted to EVs into multivesicular bodies (MVBs) and secreted via exosomes, into microvesicles shedding from the plasma membrane, or into apoptotic bodies (AB). This selective sorting towards EV subspecies has been demonstrated in Type 1 diabetes in which MCP-1 was expressed in all sorts of EVs while IL-27 was solely expressed in apoptotic bodies [86]. Fitzgerald et al. [16] observed that the number and pattern of cytokines packaged in EVs changed depending on the stimulus and suggested that the packaging of cytokines in EVs is not simply the property of a particular cytokine, but rather a tightly controlled biological process. In pathological conditions such as HIV or diabetes, it has been shown that the profile of cytokines in EVs was significantly increased and that specific cytokines were associated with disease duration [87, 88].

We may assume that in ME/CFS, inflammation may induce changes in the packing of specific cytokines/chemokines into selective EVs and that their release is intimately linked to the state of the releasing cells, healthy versus diseased, near or at a distance.

We further analyzed cytokine-cytokine interactions by performing Spearman’s correlations for each pair of cytokines in plasma and EV samples for both groups to find whether a disrupted profile of EV-associated or circulating cytokines in plasma could be identified in ME/CFS patients. Overall, there was more significant cytokine-cytokine correlations in the plasma from the ME/CFS group and less in the EV samples in comparison to healthy individuals (Figs. 4b and 5b). In ME/CFS plasma, there were no inverse correlations in the control group while there were 13 within the ME/CFS group. IP-10 was highly interconnected with 12 other cytokines in the plasma from ME/CFS (Fig. 4b) and appeared to be a negative regulator. We found IP-10 to be negatively correlated with CD40 Ligand, but this inverse relationship was not observed in a previous study [10] in which CD40 Ligand was the main driver of the inverse relationships with other cytokines. Recently, Moneghetti and colleagues found IP-10 to play a central role in the cytokine network of plasma from ME/CFS patients [11], but the IP-10 interactions with other cytokines that they reported differed from those we observed in our study population.

During neuroinflammation, NK cells and activated CD4 + and CD8 + T-cells are attracted to sites of inflammation, infected or tumoral areas in the presence of IP-10 [89]. In addition to its chemotactic properties in mediating an influx of inflammatory leukocytes into infected or inflamed tissues, IP-10 has non chemotactic functions such as inhibition of angiogenesis [90] and α-defensins-like antibacterial properties [91]. Furthermore, IP-10 has been shown to be involved in neurodegenerative disorders. In patients with Alzheimer’s disease, receptors for IP-10 are expressed on neurons and the induction of IP-10 by astrocytes and in microglia [92], along with its binding on neurons and the accumulation of beta amyloid, result in neuronal dysfunction and apoptosis [93]. In both multiple sclerosis and experimental autoimmune encephalomyelitis, IP-10 is highly expressed by astrocytes [94], and largely associated with the influx of inflammatory leukocytes into neural tissue, and disease severity. Abnormal levels of IP-10 have been observed in body fluids of individuals infected with respiratory syncytial virus (RSV) [95] and contributed to LCMV (Lymphocytic choriomeningitis virus) or West Nile Virus infections, in which IP-10 expression was restricted mainly to neurons [96]. During Herpes virus infection it has been shown that infected IP-10-deficient mice showed higher viral loads in the CNS, decreased numbers of natural killer cells and CD8^+^ T-cells [97]. Altogether, these studies highlight the important role of IP-10 in mediating CNS inflammation, a hallmark of ME/CFS, thus further focus on this chemokine is needed.

EV cytokine–cytokine correlations in ME/CFS showed a pattern that differed from controls as well, with fewer significant correlations (Fig. 5c). The network diagrams showed that both groups had a unique negative cytokine-cytokine interaction; levels of IL-1RA were inversely associated with eotaxin in the control group (Fig. 5a), and IL-7 with MCP-1 in ME/CFS EVs. Hormig et al. found an inverse relationship of IL-1RA with M-CSF, GM-CSF and IL-17 but not with eotaxin in CSF samples from ME/CFS patients [48].

Eotaxin is a member of the C–C chemokine family originally implicated in the selective recruitment of eosinophils into inflammatory sites during allergic reactions. It is induced by Th-2 cytokines, including interleukin IL-13, IL-10, and IL-4, and is produced by B cells, endothelial cells, lymphocytes, macrophages, epithelial cells, and chondrocytes [98–100]. Although it has been been thoroughly investigated in allergic reactions, eotaxin has been shown to be involved in a skewed immune response toward a type-2 (Th2) profile and was recently associated with aging, neurogenesis and neurodegeneration, being able to influence neural progenitor cells, and microglia. Increased circulating levels of eotaxin have been described in fibromyalgia [101, 102] and in several neuroinflammatory disorders [103] such as Alzheimer’s disease [104, 105], amyotrophic lateral sclerosis, Huntington’s disease, and secondary progressive multiple sclerosis [105]. It has been associated with markers of aging and degeneration and correlated with cognitive measures. Since eotaxin is capable of crossing the blood–brain barrier, it is plausible that eotaxin can exert physiological and pathological actions in the central nervous system of ME/CFS patients. Hornig et al. suggested the possibility that increased levels of eotaxin along with dysregulation of IL-1 signaling observed in ME/CFS are part of an allergic process in central compartments also seen in a CNS infections [106–108]. Altogether, eotaxin seems to be very promising to further study in ME/CFS to determine its prognostic value along with careful cognitive phenotyping monitoring and neuroimaging studies to evaluate its association with neurodegenerative changes.

Although several intercytokine network analyses were conducted by other groups in plasma and CSF [10, 11, 13, 48], there were no commonalities with our results, which may suggest again the heterogeneity of the disease and the presence of different patient subgroups. Nevertheless, our results and those of previous reports suggest unusual regulatory relationships among cytokine in plasma and EVs, indicating a disruption of intercytokine networks.

## Conclusions

The pilot study reported herein confirms previous results in which the numbers of EVs isolated from plasma of ME/CFS patients are elevated in comparison to a control group. We further analyzed the cytokine profiling in plasma and provide a unique report on the cytokine content of extracellular vesicles in ME/CFS patients. Our analysis was based on a single plasma sample from which we isolated EVs. No significant differences were found between patients and controls in both sample types. We were also not able to analyze our data based on gender nor duration of illness or disease. Even though many studies have identified potential cytokine differences between ME/CFS patients and healthy individuals, results have been contradictory (reviewed in [6–8]) and this may be due to the cross-sectional nature of the studies. Longitudinal study designs including larger numbers of ME/CFS subjects representing different clinical subgroups, and classified based on disease duration and/or severity. are needed to further characterize their association with cytokine expression.

## Supplementary information

**Additional file 1: Figure S1**. Spearman’s correlation analysis of immune analytes levels with metadata in plasma samples.

**Additional file 2: Figure S2.** Spearman’s correlation analysis of immune analytes levels with metadata in EV samples.

## Data Availability

Data for extracellular vesicle size, quantification, and cytokine content is available on request to the authors.

## Abbreviations

ME/CFS: Myalgic Encephalomyelitis/Chronic Fatigue Syndrome
EVs: Extracellular vesicles
NTA: Nanoparticle tracking analysis
TEM: Transmission electron microscopy
BMI: Body mass index
CSF: Cerebrospinal fluid
