# Supplementary figures and images for: Cytokine profiling of extracellular vesicles isolated from plasma in myalgic encephalomyelitis/chronic fatigue syndrome: a pilot study
Source: J Transl Med. 2020 Oct 12;18:387. doi: 10.1186/s12967-020-02560-0 (PMC7552484; doi:10.1186/s12967-020-02560-0)

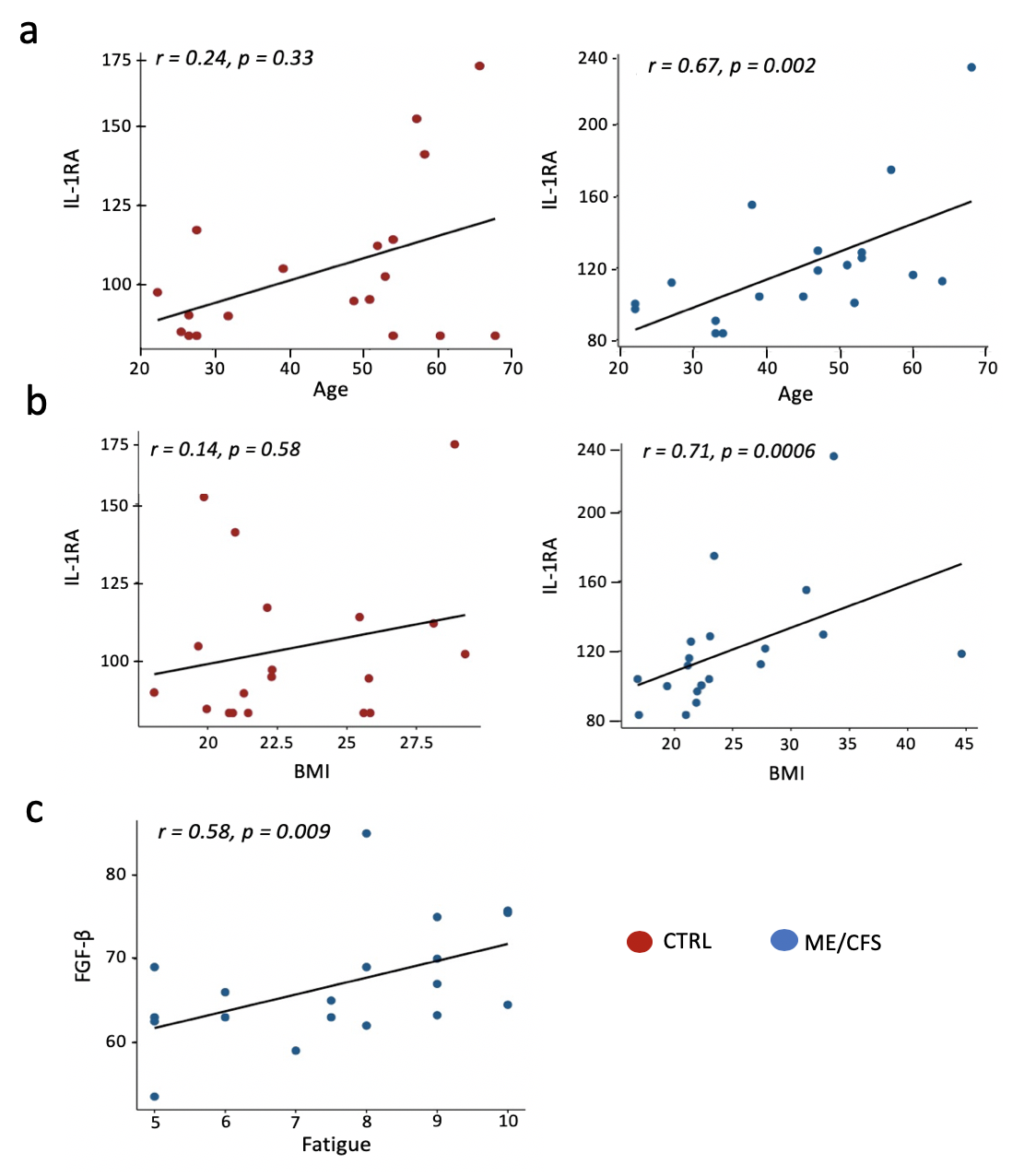

Supplement: Supplementary file 1 — Additional file 1: Figure S1. Spearman’s correlation analysis of immune analytes levels with metadata in plasma samples. [file 12967_2020_2560_MOESM1_ESM.png]

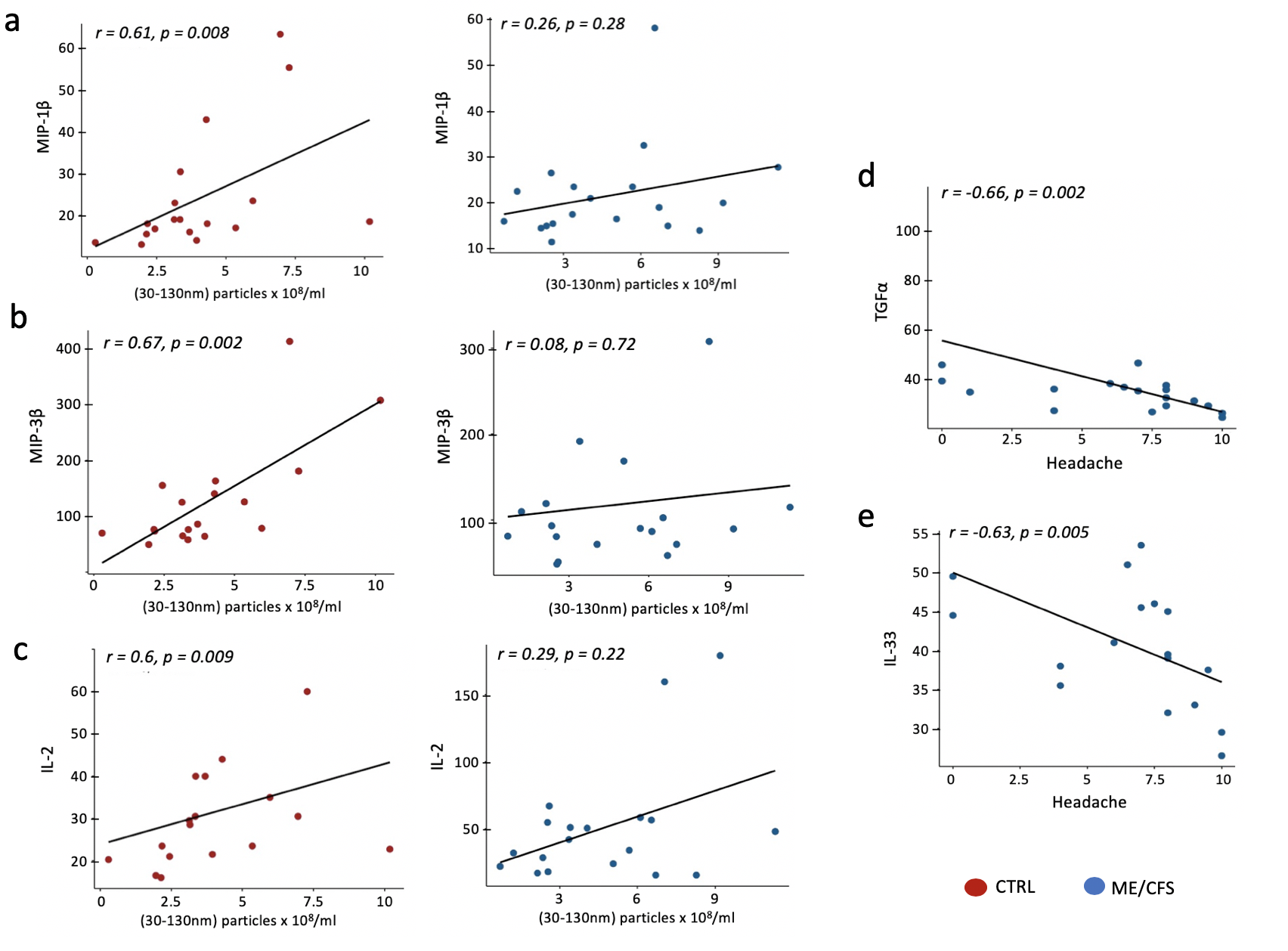

Supplement: Supplementary file 2 — Additional file 2: Figure S2. Spearman’s correlation analysis of immune analytes levels with metadata in EV samples. [file 12967_2020_2560_MOESM2_ESM.png]
